# Prediction of stroke in patients with severe aortic stenosis by left atrial appendage filling defect patterns on early and late-phase computed tomography

**DOI:** 10.1016/j.ijcha.2024.101576

**Published:** 2024-12-09

**Authors:** Pietro G. Lacaita, Sven Bleckwenn, Fabian Barbieri, Yannick Scharll, Johannes Deeg, Nikolaos Bonaros, Gerlig Widmann, Gudrun M. Feuchtner

**Affiliations:** a Department of Radiology, Innsbruck Medical University, Innsbruck, Austria; b Deutsches Herzzentrum der Charité, Department of Cardiology, Angiology and Intensive Care Medicine, Berlin, Germany; c Department of Cardiac Surgery, Innsbruck Medical University, Innsbruck, Austria

**Keywords:** Computed tomography (CT), CT-Angiography, TAVI, Left atrial appendage, Filling defects, Thrombus, Stroke

## Abstract

**Background:**

Stroke is a feared complication after TAVI. The objective was to assess whether left atrial appendage (LAA) filling-defect (FD) patterns from early and late-phase computed tomography (CT), predict stroke/TIA in patients with severe aortic stenosis.

**Methods:**

124 patients with severe aortic stenosis (79.5y, 46.8% females) who underwent CT-Angiography for TAVI-planning were included (66.1% underwent TAVI, 18.6% surgical, 15.3% conservative treatment).CT-image-analysis included: CT-density (HU) from LAA tip-to-base and HU-gradients (I-III), the HU-ratio LAA/aorta, left-atrial-wall-thickness (LAWT) and the periatrial fat attenuation index (FAI).

**Results:**

Stroke/TIA rate was 9.6 %. LAA-HU-gradient was slightly higher in non-stroke patients (p = 0.087). Persisting FDs during the late-phase were associated with stroke (p = 0.047) but not early-phase FDs. Early-phase FDs with HU < 245 (n = 15) were correlated with stroke (p = 0.05). A LAA-HU-gradient > 10HU had 91 % sensitivity and 68 % specificity for prediction of stroke. LAA-HU gradient I had a moderate accuracy (*c = 0.592;* 95 %CI:0.472–0.711; p = 0.317) for discrimination of stroke during the early phase, which enhanced during the late phase (*c = 0.686*;95 %CI:0.503–0.868; p = 0.046). Patients with stroke had a higher rate of FDs with HU-progression from early to late phase (>10HU)(p = 0.013), while the ratios LAA/aorta, LAWT, and periatrial-FAI were not different. Among clinical parameters, only age predicted stroke but not CHA2DS2-VASc-score. In multivariate analysis, late-phase FDs (p = 0.059)(OR 5.66: 95 %CI:0.936–34.28) but not early-phase FD were associated with stroke, and none of the major conventional risk factors.

**Conclusion:**

Persisting LAA-filling defects on CT during the late-phase, and early-phase FD with <245HU predict stroke, and a CT-density progression >10HU from early-to-late phase. LAA-FD may improve stroke risk stratification.

## Highlights

• Persistent LAA-filling defects during the late phase of CTA and early-phase FDs with low Hounsfield Units are the strongest predictors of stroke in patients scheduled for TAVI, performing superior to conventional risk factors and the CHA2DS-Vasc-Score.
• LAA filling defect patterns from CTA may improve stroke risk prediction in patients scheduled for TAVI over current clinical scores.
• Future research is required how to integrate LAA filling parameters from CTA into conventional risk scores.

## 1 Introduction

Transcatheter aortic valve implantation (TAVI) has emerged as a state-of-the-art treatment for severe aortic stenosis, but stroke remains one of its most feared and debilitating complications, occurring in 1.7 % to 4.8 % of cases [1], [2], [3], [4]. Despite recent reductions in the 30-day mortality rate [5], stroke rates after TAVI remain higher compared to surgical aortic valve replacement (SAVR) [6]. The incidence of silent cerebral perfusion deficits detected by MRI after TAVI is even higher [7], emphasizing the importance of stroke prevention strategies. Despite the use of embolic protection devices has decreased the risk of stroke, the reduction was minor [8]. While several strategies to prevent stroke after TAVI have been reported [9], identifying high-risk patients remains a challenge. In 2019, Thourani et al. described a risk score based on clinical risk factors, which has a moderate accuracy with c = 0.62 [10]. While the pathogenesis of stroke immediately after TAVI is supposed to be rather embolic than atherothrombotic or hemorrhagic [1], the most common source is not clear. Stroke is also a common event in all patients with severe aortic stenosis, whether they undergo surgical treatment or not, due their high age, high cardiovascular risk profile, high frailty and high burden of systemic atherosclerosis.

Left atrial thrombi are well-established risk factors for stroke [11] and mortality [12], but the role of pre-thrombotic stages like sludge with pro-coagulopathic particles accumulating in the left atrial appendage (LAA) is less clear but gaining attention for the prediction of stroke. Spontaneous echo contrast (SEC) [13] on transthoracic or transesophageal echocardiography (TEE) represents a pre-thrombotic stage, with pro-coagulopathic particles accumulating in the LAA [13]. The use of transthoracic and TEE to screen for cardiac emboli has been established in clinical practice [14].

Coronary CT- Angiography (CTA) is the modality of choice for planning of TAVI and other surgical procedures. However, distinguishing thrombi from sludge and from mere artificial filling detects (FD) due to stasis is a major challenge for CTA. During the early phase, a LAA FD with low CT density can represent both a thrombus or an artificial FD due to blood flow stasis [15], and the false positive rate of CTA is high, while late-phase CT scans reduce the rate of false-positive findings on CTA. If a FD does not resolve during the late phase, a thrombus is very likely. [16]. However, late-phase CT scans generate additional radiation and contrast volume exposure; therefore, they are not performed routinely during a CTA in patients scheduled for surgical intervention [17].

Therefore, objective of our study was to evaluate which quantitative LAA FD patterns including novel parameters (absolute Hounsfield Units (HU), gradient HU, ratio HU LAA/Aorta) during early and late phase, and other LAA morphological parameters (periatrial fat attenuation index (FAI) and left atrial wall thickness (LAWT)) from CT predict stroke in patients with severe aortic stenosis scheduled for transcatheter or surgical aortic valve intervention.

## 2 Material and methods

### 2.1 Study design and population

Patients with severe aortic stenosis who underwent electrocardiogram (ECG)-gated combined coronary and aortic CTA during 2018 and 2019 for planning of TAVI due to clinical indications were included in this retrospective single center study. Patients were scheduled for CTA after the interdisciplinary heart team, consisting of cardiac surgeons, cardiologists, radiologists, and anesthesiologists, has discussed the individual risk and appropriateness of the candidates for TAVI. Because only a limited number of procedures were permitted to be carried out per year due to financial restrictions, only very high-risk patients could be selected. Institutional review board (IRB) approval was obtained. Written informed consent was waived by IRB.

### 2.2 Inclusion criteria were

Patients with severe symptomatic aortic stenosis and a high cardiac operative risk (including those with 20 % or more), meeting the criteria for surgical intervention [18], were selected as potential candidates for TAVI by our interdisciplinary heart team and scheduled for CTA. Diagnosis of severe aortic stenosis was made by transthoracic echocardiography according to standardized criteria [18]. **Exclusion criteria** for CTA were: severe renal dysfunction (glomerular filtration rate (GFR) < 30 ml/min), untreated hyperthyreosis, prior iodine contrast allergy without adequate premedication and pregnancy. For TAVI, exclusion criteria were active endocarditis, myocardial infarction within 14 days, cardiogenic shock, and a life expectancy of less than 1 year.

Major **traditional cardiovascular risk factors (CVRF)** were collected and defined according to standardized European Society of Cardiology (ESC) criteria arterial hypertension (systolic blood pressure (BP) > 140 mmHg or diastolic BP > 90 mmHg), dyslipidemia, positive family history (myocardial infarction (MI) or sudden cardiac death in an immediate male relative < 55 years or female < 65 years), smoker (active: current or quit less than 6 months before coronary CTA examination and former), and diabetes [19], [20], [21]. Further clinical parameters, all co-morbidities (including coronary artery disease, myocardial infarct, prior stroke), stroke event details (TIA vs stroke), CHA2DS2-VASc score, and medications were collected. All data were retrieved from our internal hospital documentation system, and recorded by board-certified physicians.

### 2.3 Computed tomography (CT)

A 128-slice dual-source CT (*Definition FLASH or DRIVE, Siemens Healthineers, Erlangen, Germany*) scanner with a detector collimation of 2 × 64 × 0.6 mm and a rotation time of 0.28 s was used. Scans were triggered into the arterial phase using bolus tracking (threshold of 100 HU, ascending aorta) and by injecting an intravenous iodine contrast agent (*Iopromide, Ultravist 370*™, Bayer Healthcare, Berlin, Germany*)*. A 2-step CT scan protocol was deployed: First, a CT-Angiography scan during arterial phase (“early phase”) was performed: A retrospective ECG-gated helical CTA of the heart with image acquisition during both systolic and diastolic phases was acquired with a bolus of 60–110 ml iodine contrast agent. The amount of contrast agent was adjusted to a patient́s individual body weight using a standardized regimen. The CT scan was triggered into the arterial phase (ascending aorta, threshold 100 HU) by using automated bolus tracking. Second, a CT-Angiography scan during late phase was performed after a delay of over 5 min. Prospective ECG-triggered CTA covering the entire thoracic and abdominal/pelvic scan for the evaluation of vascular access routes was conducted with high-pitch mode (pitch, 3.2), triggered into diastolic phase (60 % of RR-interval) and into arterial phase, as outlined above.

### 2.4 CT image postprocessing

Axial thin slice images were reconstructed with a 1.0 mm slice width (increment, 0.7) and transferred to clinically approved radiology software (DeepUnity Diagnostic 1.2.0.1, Dedalus, DH Healthcare, Bonn, Germany). The following measurements were taken:

### 2.5 CT image analysis

**Fig. 1:**
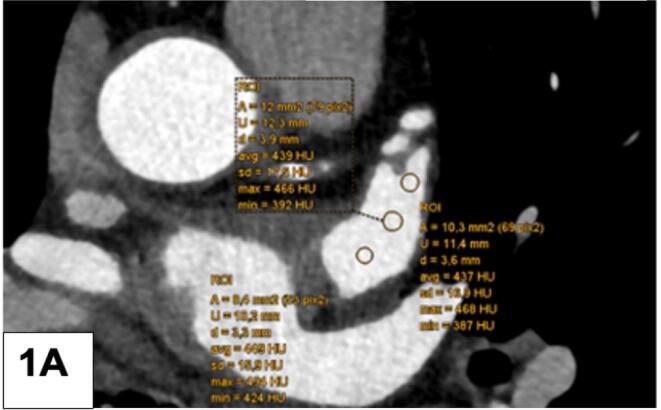

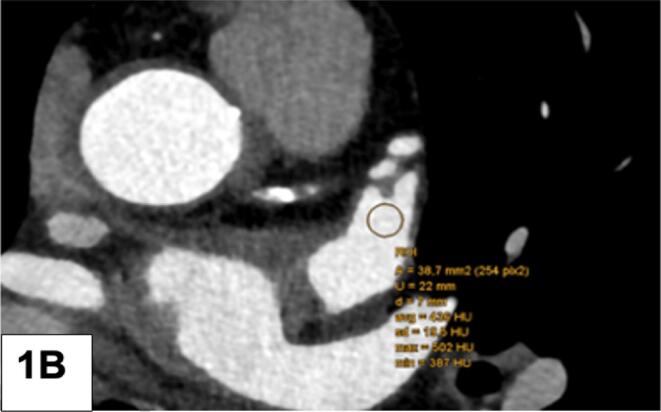
A LAA CT density (HU) quantification: 3 regions of interest (ROI) were placed from LAA tip to the bottom and the gradient was calculated. A normal LAA, entirely filled with contrast agent, is shown. B: Mean LAA HU density of a normal LAA: A large ROI was placed into the LAA.

**Fig. 2:**
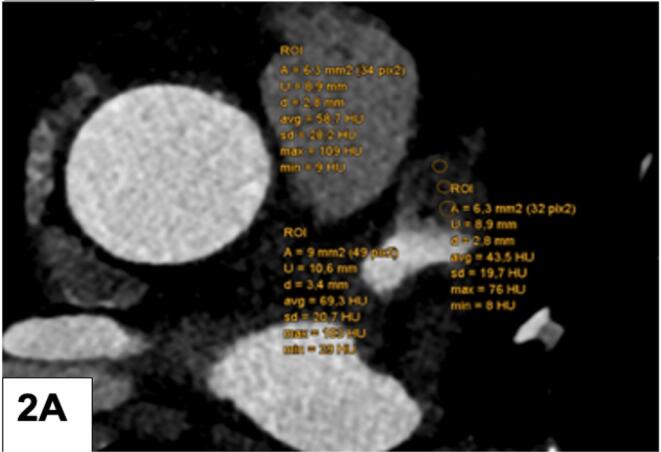

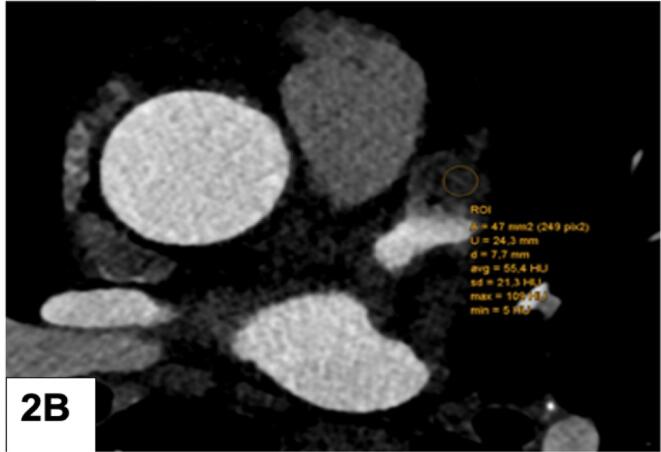
A: LAA filling defect (FD): Measurement of the HU gradient from the tip to the bottom: 3 ROI were drawn from LAA tip to the bottom. HU 1 (top), HU 2 (mid section), HU 3 (bottom) B: Mean HU of the LAA FD was quantified by placing one large ROI.

**Fig. 3:**
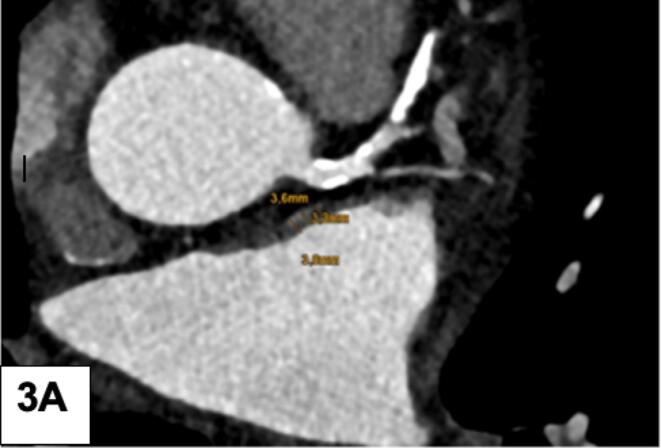

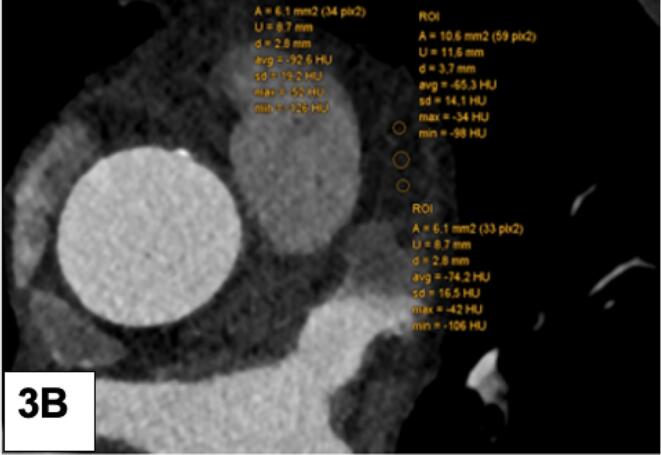
A: Left atrial wall thickness (LAWT) was measured at 3 different sites along the left atrial ridge. B: The periatrial fat attenuation index (FAI) was measured adjacent of the tip of the LAA.

First**,** the CT density of the LAA (HU) was measured by locating the left atrial appendage (LAA) and placing three round-shaped region of interest (ROI) from the tip to the bottom (apex/mid/basal) without filling defect (Fig. 1a – normal LAA filled with contrast agent) and a filling defect (Fig. 2a), and by placing one large ROI centrally in the left atrial appendage (normal, filled with contrast agent) without filling defect (Fig. 1b) and similarly, into a LAA filling defect (Fig. 2b). A filling defect was defined as a hypoattenuating region within the LAA, not filled with contrast agent. The diameter of the filling defect (FD) was measured with a virtual caliper. Second, the ratio of the CT density of the LAA to the ascending aorta (HU) was calculated after placing a large ROI into the ascending aorta. Third, left atrial wall thickness (LAWT) was measured at three different sites using the virtual ruler (Fig. 3a) along the anterior left atrial ridge. The CT slice was located approximately at the bifurcation of the left coronary artery (LCA) into the circumflex artery (CX) and the left anterior descending (LAD). Forth, the periatrial fat attenuation index (FAI) was measured by placing three different ROIs arranged in a row adjacent to the tip of the LAA (Fig. 3b). The same measurements were repeated in the corresponding late-phase protocol (if available) by one experienced observer. Three different LAA HU gradients were calculated as follows: gradient I (between ROI 1 (tip) and ROI 3 (bottom)), gradient II (between ROI 1 (tip) and ROI 2 (mid)), and gradient III (between ROI 2 (mid) and ROI 3 (bottom)). A “persisting FD” was defined as a lack of LAA filling during late phase. Further, the LAA HU progression from early to late phase was calculated as a novel quantitative LAA parameter for prediction of stroke.

### 2.6 TAVI procedure, surgical technique

TAVI was performed in hybrid operating rooms equipped with an invasive angiography suite by an interdisciplinary heart team consisting of both cardiac surgeons and interventional cardiologists. A transfemoral or transaxillary access route was chosen. Prosthesis sizing was based on CTA annulus dimensions. TAVI was conducted with transesophageal and intracardiac echocardiography under fluoroscopic guidance, and with general anesthesia.

### 2.7 Outcomes

Cerebrovascular events, were defined as either a stroke (diagnosed with clinical and imaging (CT/MR) criteria) in accordance with the recommendation by the Academic Research Consortium Initiative [22], or transient ischemic attack (TIA)- defined as neurological deficit lasting ≥24 h or lasting <24 h with a brain imaging study (MRI and/or CT) providing evidence.

### 2.8 Statistical analysis

Statistical analysis was performed using SPSS™ software (V25.0, SPSS Inc., Chicago, USA). Quantitative variables are expressed as means ± standard deviation (SD) or as median (IQR), according to their distribution, and categorical variables as absolute values and percentages. Normal distribution of data was tested with Kolomogorov-Smirnow test and histogramm. Chi-square test was applied to test for differences in categorical data. Independent *t*-test was applied to test for differences in parametric and normally distributed data (such as age, body mass index (BMI), and CT LAA imaging data) among stroke and non-stroke patients.

Receiver Operating Curves (ROC) were generated to define the diagnostic accuracy (c-value) of all quantitative CT imaging LAA parameters listed in the methods for prediction of stroke. Univariate and multivariate binary regression analysis were generated for prediction of stroke by CT imaging parameters, the major cardiovascular risk factors and the CHA2DS-VASc-Score.

## 3 Results

This retrospective study included 124 patients, 46.8 % of whom were female (Table 1). Out of 124 patients with severe aortic stenosis, 66.1 % underwent TAVI, 18.6 % underwent surgical treatment, and 15.3 % received conservative treatment (Table 2). The average age was 79.4 ± 7.1 years, with a range of 52 to 89 years. Table 1 shows the study cohort profile, including differences between stroke patients and those who did not have a stroke.

**Table 1:** Study cohort: patient profile (n = 124).

	**n = 124**	**Stroke (n = 12)**	**No Stroke (n = 112)**	**p-value**
**Age (years)**	79.4 ± 7.1	83.3 ± 5.5	79.0 ± 7.2	0.023*
**BMI (kg/m^2^)**	26.2 ± 4.9	24.3 ± 5.1	26.4 ± 4.8	0.196
**Female (%)**	58 (46.8)	6/12 (50)	52/112 (46.4)	0.81
**Arterial Hypertension (%)**	88/124 (71.0)	8/12 (66.7)	80/112 (71.4)	0.73
**Nicotine Abuse (%)**	31/124 (25)	1/12 (8.3)	30/112 (26.8)	0.16
**Family History (%)**	40/124 (32.3)	5/12 (41.7)	35/112 (31.3)	0.46
**Dyslipidemia (%)**	88/124 (71.0)	7/12 (58.3)	81/112 (72.3)	0.31
**Diabetes (%)**	28/124 (22.6)	1/12 (8.3)	27/112 (24.1)	0.21
**Obesity (%)**	22/124 (17.7)	1/12 (8.3)	21/112 (18.8)	0.37
**Pre-obesity (%)**	46/124 (37.1)	7/12 (58.3)	39/112 (34.8)	0.11
**Typical chest pain(%)**	42/124 (33.9)	3/12 (25)	39/112 (34.8)	0.49
**History of Myocardial Infarction (%)**	19/124 (15.3)	3/12 (25)	16/112 (14.3)	0.33
**History of Stent/PCI (%)**	34/124 (27.4)	6/12 (50)	28/112 (25)	0,066
**History of CABG (%)**	13/124 (10.5)	1/12 (8.3)	13/112 (11.6)	0.021*
**History of Stent & CABG (%)**	5/124 (4.0)	0/12 (0)	5/112 (4.46)	0,455
**Atrial Fibrillation (%)**	58/124 (46.8)	7/12 (58.3)	51/112 (45.5)	0.27
**Aspirin (%)**	77/124 (62.1)	9/12 (75)	68/112 (60.7)	0.33
**NOAC (%)**	30/124 (24.2)	2/12 (16.7)	28/112 (25)	0.52
**DAPT (%)**	19/124 (15.3)	1/12 (8.3)	18/112 (16.1)	0.48
**Vitamin K Antagonists (%)**	9/124 (7.3)	1/12 (8.3)	8/112 (7.1)	0.88
**Pacemaker (%)**	16/124 (12.9)	1/12 (8.3)	15/112 (13.4)	0.62

Parametric variables are expressed as means ± standard deviation (SD), categorical variables as absolute values (N) and percentages (%). BMI = body mass index. CABG: Coronary artery bypass grafting. NOAC = novel oral anticoagulant DAPT = Dual antiplatelet therapy.

**Table 2:** Procedure related results.

	**n = 124**
**Age (years)**	79.4 ± 7.1
**CHA2DS2-VASc-Score**	4.05 ± 1.7
**TAVI (%)**	82 (66.1)
**SAVR (%)**	23 (18.5)
**Conservative (%)**	19 (15.3)
**Stroke Rate (overall) (%)**	12/124 (9.6)
**Stroke Rate (TAVI) (%)**	12/82 (14.6)
**Stroke Rate (SAVR) (%)**	0/23 (0)
**Pre-TAVI Events (%)**	17/124 (13.7)
**CTA to OP (days)**	62.7 ± 82.8
**CT (Early- % Late phase) (%)**	77/124 (62.1)
**Filling Defects (Early phase) (%)**	n = 25 (20.2)
**Filling Defects (Late phase) (%)**	n = 10 (8.1)
**Filling Defects (both phases) (%)**	n = 10 (8.1)
**Filling defect disappeared in 2nd phase (%)**	11/25 (44)

Abbreviations: TAVI: Transcatheter aortic valve replacement, SAVR: Surgical Aortic Valve Replacement.

The mean CHA2DS-VASc-Score was 4.05 ± 1.7 (range, 0–9). There was no statistically significant difference in cardiovascular risk factors between stroke patients and those who did not have a stroke. Only age (p = 0.023) was higher in stroke patients, while BMI was not different. EuroScore II was 15.23 +/- 3.1 SD.

Additionally, 50 % of the stroke group had a previous intervention with coronary stents, which was not different from the event-free group (p = 0.066). The event-free group had more patients who underwent coronary artery bypass grafting (CABG) (p = 0.021). CTA revealed no difference in the proportion of those with obstructive coronary artery disease (>50 % coronary stenosis) between stroke and non-stroke patients (77.8 % vs 72.9 %, p = 0.753). The prevalence of atrial fibrillation was similar between the groups (58.3 % vs. 45.5 %, p = 0.27). Ten of the 16 patients with pacemakers were placed during or shortly after the intervention. There were no significant differences between the groups in terms of continuous anticoagulant drug use.

Clinical outcomes: Table 2 shows outcomes, treatment, and CT results of the entire cohort: The time interval between CT and surgical intervention was on average 62.8 ± 82.84 days (range, 3 to 514 days). The cerebrovascular event rate during the observed period was n = 12 (9.6 %). Nine stroke events (75 %) and n = 3 TIA (25 %) were recorded. Of nine strokes, n = 1 died related to a large medial cerebral artery territorial infarct, followed by coma and death. There were n = 3 medial cerebral artery (MCA) partial or complete territorial infarcts (of which n = 1 also had additional embolic lesions), 1 patient with both medial cerebral artery infarct and posterior infarct right, n = 1 infarct with intracerebral hemorrhage (ICH), n = 2 posterior/occipital infarcts, n = 1 small cortical frontoparietal, and n = 1 subcortical embolic stroke events. All TIA events were cardioembolic and verified by MRI. Symptoms of patients with TIA were: n = 1 hemiparesis and aphasia, and n = 2 typical MCA TIA related symptoms. The 12 Stroke events occurred in the TAVI group only (100 %), while no strokes (0 %) were recorded in the SAVR or conservatively treated groups. Time from CT to the stroke event was mean 206.5 days ± 275 SD (range, 0–792 days). There were only 3 events (25 %) within 30 days (short term) and 9 (75 %) events occurred during long- term follow up (>=30 days).

Left atrial appendage filling defects (LAA) patterns by CTA: Early-phase CTA protocols were available for all 124 patients, and 62.1 % had a corresponding late-phase protocols. Among the 124 early-phase CTAs, a filling defect (FD) was observed in 25 cases (20.2 %). Of these, 10 FDs (40 %) persisted in the late phase, while 11 (44 %) resolved (artificial filling defects). In 4 cases, an early-phase FD could not be followed up due to the lack of a late-phase CT (additional contrast could not be administered due to renal dysfunction). Table 3a shows the quantitative CT parameters of LAA FDs during early and late phases. Persisting FD during late phase had a significantly higher CT density of 233.19 HU compared to early arterial FDs (p = 0.004). Persisting FDs during late-phase were associated with stroke (p = 0.047) on univariate binary regression but not early-phase FD (Table 3b). Early-phase FD with less than 245 HU (n = 15) were correlated with stroke (p = 0.05), but not those with higher HU above 245 HU.

**Table 3a:** Quantitative CT-Parameters of LAA filling defects (FD): absolute values.

	**Early Phase (n = 25)**	**Late Phase (n = 10)**
**Filling Defects (%)**	25/124 (20.2)	10/77 (13)
**Size of Filling Defects (mm)**	19.8 ± 6.5	20.2 ± 7.3
**Ascending aorta (HU)**	433.6 ± 82.8	386.7 ± 86.8
**LAA 1 (HU)**	124.8 ± 97.6	219.0 ± 119.8
**LAA 2 (HU)**	137.1 ± 99.7	230.9 ± 113.1
**LAA 3 (HU)**	156.4 ± 108.4	249.7 ± 112.7
**Sum of HU 1**–**3 (mean)**	139.4 ± 98.7	233.2 ± 113.3
LAA Mean (HU)*	136.2 ± 91.2	222.7 ± 112.2

*one large ROI.

Abbreviations: HU = Hounsfield Units. LAA = left atrial appendage.

**Table 3b:** Univariate binary logistic regression testing for prediction of stroke: Among FD, only persisting FD during late phase showed a significant association with stroke, but not early phase.

	**OR**	**95 % CI**	**p-value**
**Persisting FD during late phase**	5.229	1.023 – 26.716	0.047*
**FD during early phase**	2.167	0.596––7.876	0.240
**FD disappeared during late phase**	4.286	0.094–9.249	0.246
**CHA2DS2-VASc-Score**	1.353	0.939–1.951	0.105

Table 4 shows quantitative CT-density (HU) values of the LAA and HU gradients during early and late phase. LAA HU-gradient I was slightly higher in non-stroke patients, although not statistically significant. (50.6 HU vs 34.9 HU; p = 0.087), while all other gradients I-III were not different. On ROC analysis, among the different HU Gradients I, II, and III during early phase, HU Gradient I had the highest accuracy (c = 0.592; 95 % CI: 0.472–0.711; p = 0.317) for prediction of stroke (Supplement 1), as compared to gradients II and III (c = 0.358; 95 %CI: 0.192–0.523; p = 0.120 and c = 0.544; 95 %CI: 0.372–0.716, p = 0.632). Similarly, during late phase, Gradient II and III had a lower accuracy (c = 0.543; 95 % CI: 0.392–0.695, p = 0.575, and c = 0.583; 95 % CI: 0.316–0.851, p = 0.542) compared to gradient I (c = 0.686; 95 % CI: 0.503–0.868; p = 0.046), which was the only significantly predictor of stroke (Supplement 1). A LAA gradient of > 10HU had the highest (91 %) sensitivity and 68 % specificity for prediction of stroke. Finally, patients with stroke had a higher rate of FDs with HU progression (>10HU) from early to late phase (25 % vs 5.4 %; p = 0.013).

**Table 4:** LAA HU-Gradients during early and late CT phase in all patients, including those with normally filled LAA: Between patients with and without stroke, no significant differences were found.

**Early phase**				
	**Stroke (n = 12)**	**No Stroke (n = 112)**	**Total (n = 124)**	**2-sided p-value**
**HU 1 (Top)**	342.8 ± 200.6	361.0 ± 162.4	359.3 ± 165.2	0.919
**HU 2 (Mid)**	349.2 ± 191.5	362.6 ± 153.2	361.4 ± 156.1	0.825
**HU 3 (Bottom)**	349.8 ± 189.9	372.5 ± 151.8	370.5 ± 154.8	0.972
**HU (Mean)**	347.3 ± 193.3	364.5 ± 154.3	362.9 ± 157.3	0.926
**Gradient I** **(HU 3**–**1)**	29.5 ± 13.3	32.1 ± 35.1	31.8 ± 33.7	0.316
**Gradient II** **(HU 1**–**2)**	16.6 ± 15.0	28.3 ± 29.8	27.2 ± 28.9	0.120
**Gradient III (HU 2**–**3)**	23.3 ± 24.5	25.4 ± 24.5	24.1 ± 29.7	0.632

**Late phase**				
**HU 1 (Top)**	273.0 ± 152.4	348.3 ± 151.0	340.5 ± 151.9	0.278
**HU 2 (Mid)**	285.9 ± 138.9	349.0 ± 147.3	342.4 ± 146.8	0.452
**HU 3 (Bottom)**	316.1 ± 126.0	349.2 ± 142.2	345.8 ± 140.2	0.658
**HU (Mean)**	291.7 ± 137.9	348.8 ± 145.0	342.9 ± 144.5	0.483
**Gradient I** **(HU 3**–**1)**	50.6 ± 26.7	34.9 ± 28.6	36.5 ± 28.6	0.087
**Gradient II** **(HU 1**–**2)**	26.4 ± 10.1	30.6 ± 26.9	30.2 ± 25.7	0.689
**Gradient III (HU 2**–**3)**	37.3 ± 34.7	24.9 ± 21.4	26.2 ± 23.2	0.442

The ratio of the CT-density (HU) in the LAA/aorta was not different between stroke and non-stroke patients (−91.8HU vs. 83.7HU; p = 0.190), and the accuracy for both early and late phases was moderate (c = 0.531, 95 % CI: 0.314–748, p = 0.736 for early phase) and poor (c = 0.402, 95 % CI: 0.192–613, p = 0.367 for late phase), respectively.

### 3.1 Left atrial wall thickness (LAWT) and periatrial fat attenuation index (FAI)

Left atrial wall thickness (LAWT) and the periatrial fat attenuation index (FAI) were not different between stroke and non-stroke patients (LAWT: mean 3.2 mm ± 0.83 vs 3.5 ± 0.69, p = 0.117 and periatrial FAI: mean −83.7 ± 20.67 vs −91 ± 19.7, p = 0.175, respectively).

### 3.2 Clinical and cardiovascular risk factors (CVRF) compared to LAA FD patterns by CTA

**Fig. 4:**
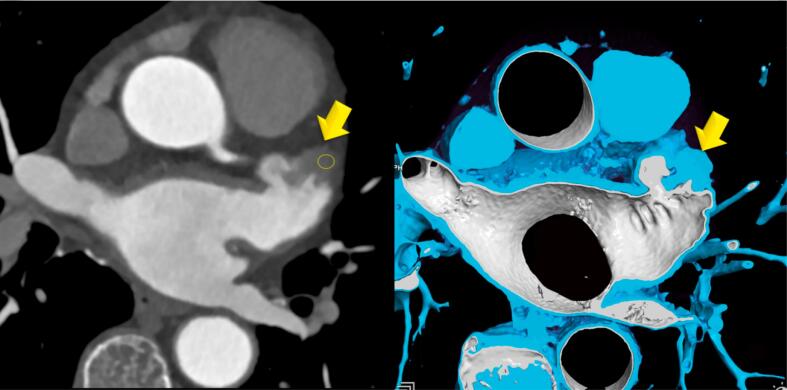
Case example of a LAA FD during early phase with 52 HU, representing a thrombus in a 76 years old male patient with atrial fibrillation.Left: MPR, and right: Thin slice volume rendering technique (VRT).

On multivariate analysis, FD during late phase (p = 0.059) (OR 5.66: 95 %CI: 0.936–34.28) but not during early-phase were borderline associated with stroke, but none of the conventional major risk factors (Table 5). Univariate testing revealed only age (OR 1.3: 95 %CI: 1.041–1.610) being associated with stroke but not CHA2DS2-Vasc-Score. Fig. 4 shows a 76-year-old male patient with atrial fibrillation and a FD in the LAA with 52 HU, representing a thrombus.

**Table 5:** Binary logistic regression analysis: Association of the major cardiovascular risk factors (CVRF) with stroke, and filling defects (FD) during late phase: only LAA filling defects (FD) during late phase showed a borderline association (p = 0.059) with a high OR of 5.66.

**CVRF**	**OR**	**95 % CI**	**p-value**
smoking	0.471	0.044–4.995	0.532
arterial hypertension	3.695	0.473–28.873	0.213
dyslipidemia	0.419	0.071–2.474	0.337
diabetes	0.000	0–0	0.998
obesity	0.521	0.040–6.812	0.619
FD late phase	5.666	0.936–34.280	0.059
positive familiy history	0.821	0.138–4.880	0.828

## 4 Discussion

Our primary study findings are as follows: Cerebrovascular events can be predicted using pre-interventional identifiable LAA-FDs patterns on CTA in patients undergoing TAVI planning, particularly if the patterns continue into the late phase. The rates of stroke/TIA after TAVI and persistent LAA-FD during late phase were significantly correlated in our study. Early-phase FD, on the other hand, did not exhibit a similarly robust association. Therefore, the late-phase FDs may be more accurate and significant indicators of stroke risk than transitory stasis artifacts since they more likely show the existence of thrombi rather than mere artifacts from lack of contrast agent mixing with blood during early phase. Importantly, out of 124 patients with severe aortic stenosis, 66.1 % underwent TAVI, 18.6 % underwent surgical treatment, and 15.3 % received conservative treatment – so our cohort is a mixed and heterogenous cohort.

So far, only one single-centric study has examined LAA-FD patterns for the prediction of stroke following TAVI. According to Okuno et al. [23], patients with LAA-FDs had higher rates of stroke after TAVI (9.2 %) (P < 0.001) and a higher history of cerebrovascular events (16.4 % vs. 10.9 %, P = 0.045) [23]. Patients with a definite thrombus had the highest risk of disabling stroke (24.0 %), followed by a “HU-run-off” (8.0 %) in the LAA, a pattern that was similar to the HU-gradient we observed in our study. However, the authors evaluated only early and not late phase CT scans [23].

Increased stroke risk is associated with the presence of thrombus or blood stagnation in the left atrial appendage (LAA) [24] and LAA-FDs on CTA represent imaging markers for this condition. However, LAA-FDs during the early phase are less specific because they rather often reflect a lack of contrast agent filling during the early phase due to flow stasis due to the supine position of the patient. However, early phase FD may contain thrombi or sludge, which resolve later because the complete mixing of iodine contrast agent with blood in the systemic circulation takes several minutes. Therefore, during late CT phase, an artifact due to stasis is resolved, while a thrombus continues to appear hypodense with low CT attenuation values [25], [26]. Definite left atrial appendage thrombi have been reported as predictors of stroke after TAVI by van Wiechen et al. [11]. Another study by Szekely et al. [12] found no difference in the short-term (in-hospital) cardiovascular event rate but observed an influence of LAA thrombi on mortality, a potential late complication of stroke occurring during long-term follow-up.

Further, we performed a quantitative HU analysis of LAA-FD from tip to the base, revealing that an LAA HU gradient greater than 10 HU is another predictor of stroke in patients scheduled for TAVI, with high sensitivity (91 %) and moderate specificity (68 %) even during the early phase. During the late phase, the HU gradient's predictive power was improved. The non-stroke group exhibited a trend toward higher values in the LAA HU gradient between the tip and base, possibly pointing at a higher probability of mere artificial filling defects due to flow artifacts, rather than the presence of SEC or LAA thrombi. According to a ROC analysis, there was moderate discrimination capacity in the early phase (C = 0.592), but this improved in the late phase.

Third, we describe another novel CT imaging biomarker, the LAA HU progression from early to late phase. This parameter has not been documented in the literature so far. Patients with stroke had a higher rate of FDs with HU progression from early to late phase (>10 HU). This imaging biomarker may improve the accuracy of stroke risk prediction following TAVI.

Forth, the “ratio HU LAA/aorta” has been previously reported as a useful criterion to rule out LAA thrombi. In our cohort, the stroke group showed a tendency towards a lower ratio throughout both the early and late CT phases, however this trend did not reach a statistical significance in our cohort. Patel et al. previously described a cut-off of 0.75 as a criterion for differentiating artificial filling defects to exclude thrombi [27]. Overall, the accuracy of CT for exclusion of thrombi is high [26] if the LAA is completely filled with contrast [16]. However,FDs in the LAA, especially during the early phase, are causing a high rate of false positive findings [26] because they can represent either thrombi, sludge, or artificial filling detected due to stasis that usually resolve during the late phase. Adding a late phase improves the accuracy of CT. [25].

Compared to early-phase FDs (139.42 HU), persistent filling defects had a considerably greater density of 233.19 HU. Furthermore, cerebrovascular events following TAVI were predicted by early-phase FD with a lower HU of <245 HU. Potential causes may include a comparatively high sludge content (“spontenaous echo contrast, SEC” on echocardiography) [13] compared with thrombi that are consistently less dense in both the early and late phases (without increased density). Additionally, a longer circulation time following contrast agent injection and the presence of an artificial FD during the early phase may contribute to an increased filling of the LAA with iodinated contrast medium in the late phase. As it can be difficult to distinguish between the two, we measured the HU in the LAA at three separate locations (the tip, midsection, and bottom) and computed three distinct gradients, of which of which the gradient I (HU difference between the tip and the bottom of the LAA) had the highest diagnostic accuracy for the prediction of stroke.

Our findings showed that traditional clinical risk factors such as hypertension, diabetes, and prior stroke did not significantly predict stroke incidence in our cohort. Stroke patients were older, and severe coronary artery disease (CAD) was more prevalent among stroke patients. However, multivariate analysis indicated that only late-phase LAA-FDs had a more pronounced association (OR 5.66) with stroke risk than these conventional factors and CHA2DS2-VASc-score. Therefore, using quantitative LAA-FD analysis may improve the accuracy of stroke prediction over the existing clinical risk assessments that rely on traditional risk variables such as the clinical risk score described by Thourani et al. (2019), which had moderate accuracy [10]. However, more specific combined model ROC analysis are required, to test whether LAA-FD add incremental value to existing risk scores.

Further, left atrial wall thickness (LAWT) did not significantly differ between the stroke patients and the control group. Other studies have reported LAWT as a predictor of stroke [28]. However, patients with more severe left atrial remodeling as compared to our cohort were included in prior studies [28]. LAWT was only mild (mean 3 mm) in our patients, which may explain why LAWT was not a prognosticator. As well, the *peri*-LAA fat attenuation index (FAI) showed no significant difference between the stroke/TIA and control group. Epicardial adipose tissue (EAT) volume may predict stroke, as reported in a *meta*-analysis pooling eight studies [29]. This discrepancy may be explained by the low sample size in our study and by the distinct measurement technique applied in our cohort. We calculated only the periatrial FAI but not the entire epicardial fat volume.

Finally, of note, despite the conventional clinical risk score for cerebrovascular events (CHA2DS2-VASc) was with 4.05 rather high in our cohort, it was overall not predictive for stroke. This result highlights the potential of CT-based imaging biomarkers to improve the precision of stroke and TIA event prediction [22] following TAVI. Further, the composite cerebrovascular event rate consisting of both stroke and TIAs was rather high in our cohort compared to existing literature. This can be attributed to the very high-risk profile of our cohort, which include advanced age, elevated surgical and cardiovascular risk, and a high incidence of atrial fibrillation. Beyond, the inclusion of TIAs, which accounted for 25 % of all events contributed to the total high event rate.

Of note, nowadays, also patients with intermediate surgical risk are recommended for TAVI according to novel guidelines. Therefore, our very high risk cohort is not representative for intermediate risk patients who are nowadays selected for TAVI.

Limitations. Although the sample size of our study population is rather small, it is sufficiently powered, with an overall stroke rate of 9.6 %. Stroke rate was high due to the cohort profile (only very high risk patients were included). Second, interobserver variability was not calculated. Third, stroke events occurred both during short and long-term follow up (> 30 days), with the majority (75 %) after 30 days. Stroke mechanism may vary, and rather procedure related during short-term follow-up. Further, changes in medication during the follow-up period may affect stroke risk, but were not recorded. Furthermore, a bias could have been introduced because the impact of various antithrombotic medication regimens on stroke was not investigated. Different antithrombotic medication regimens are influencing outcomes after TAVI [12], other surgical procedures and conservative treatment.

## 5 Conclusion

Our study demonstrates that persistent LAA-FDs during the late phase of CTA and early-phase FDs with low HU values are promising predictors of stroke in patients with severe aortic stenosis scheduled for surgical interventions. Identifying patients at risk for cerebrovascular events is a major challenge, and quantitative LAA-FD parameters could be useful to sharpen risk prediction. Future research to validate these findings in larger, external, or preferably multicentric cohorts would be desirable. In particular, the incremental value of LAA-FD patterns over conventional clinical risk scores for prediction of cerebrovascular events requires further investigations.

## CRediT authorship contribution statement

**Pietro G. Lacaita:** Writing – reviewing & editing, Data curation. **Sven Bleckwenn:** Writing – review & editing, Data curation. **Fabian Barbieri:** Writing – review & editing, Validation, Investigation. **Yannick Scharll:** Writing – review & editing, Data curation. **Johannes Deeg:** Writing – review & editing, Data curation. **Nikolaos Bonaros:** Writing – review & editing, Data curation. **Gerlig Widmann:** Writing – review & editing, Resources. **Gudrun M. Feuchtner:** Writing – review & editing, Writing – original draft, Methodology, Data curation.

## Declaration of competing interest

The authors declare that they have no known competing financial interests or personal relationships that could have appeared to influence the work reported in this paper.
