# Supplementary material for: Prediction of stroke in patients with severe aortic stenosis by left atrial appendage filling defect patterns on early and late-phase computed tomography
Source: Int J Cardiol Heart Vasc. 2024 Dec 9;56:101576. doi: 10.1016/j.ijcha.2024.101576 (PMC11681882; doi:10.1016/j.ijcha.2024.101576)

**Supplement 1a.** **Accuracy of LAA FD patterns for prediction of stroke:** ROC curve for early phase (HU gradient I) *c=0.592;* 95%CI:0.472-0.711; p=0.317


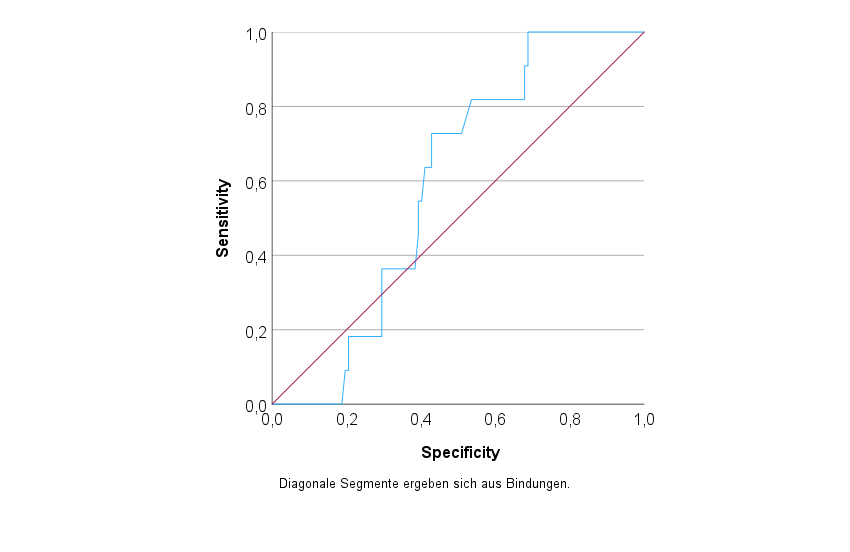


**Supplement 1b.** ROC curve for late phase FD: *c=0.686*; 95%CI:0.503–0.868; p=0.046


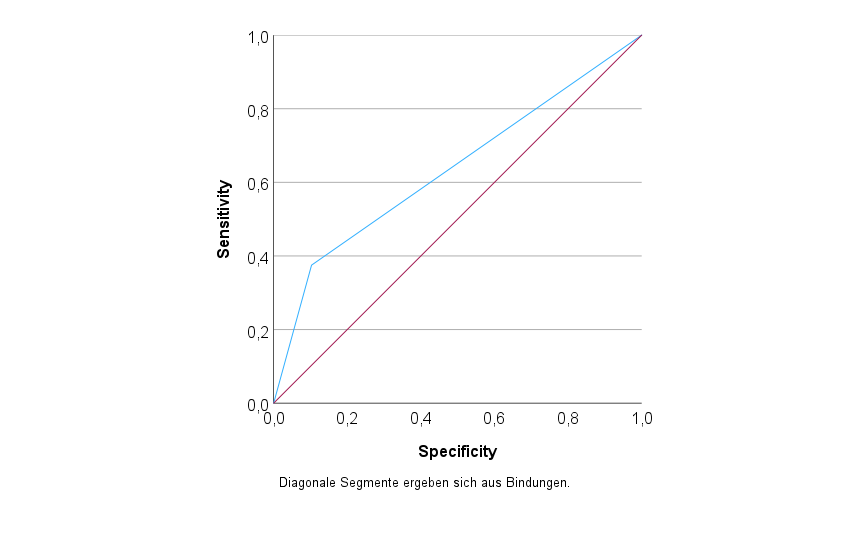

Supplement: Supplementary Data 1 [file mmc1.docx]
